# Innovative prototypes for cervical cancer prevention in low-income primary care settings: A human-centered design approach

**DOI:** 10.1371/journal.pone.0238099

**Published:** 2020-08-24

**Authors:** Marcela Arrivillaga, Paula C. Bermúdez, Juan Pablo García-Cifuentes, Jorge Botero

**Affiliations:** 1 Department of Public Health and Epidemiology, Pontificia Universidad Javeriana, Cali, Colombia; 2 Department of Electronics and Computer Science, Pontificia Universidad Javeriana, Cali, Colombia; 3 Centro de Consultoría y Educación Continua, Pontificia Universidad Javeriana, Cali, Colombia; City, University of London, UNITED KINGDOM

## Abstract

This article presents the design process of innovative prototypes for cervical cancer prevention in primary care centers located in low-income settings in Cali, Colombia, using the Human-Centered Design (HCD). The project was developed in collaboration with a public healthcare network comprised of 38 urban and rural centers with women between the ages of 25 and 65 years, healthcare providers of the cancer program, healthcare administrators and the general manager of said network. Our HCD process involved five stages: research, need synthesis, ideation and co-design process, prototyping and in-context usability testing. In practice, some of the stages are overlapped and iterated throughout the design process. We conducted observations, open-ended interviews and conversations, multi-stakeholder workshops, focus groups, systematic text condensation analyses and tests in real contexts. As a result, we designed four prototypes: (1) ‘Encanto’: An educational manicure service, (2) ‘No le des la espalda a la citología’: A media-based strategy, (3) An educational wireless queuing device in the waiting room, and (4) Citobot: A cervical cancer early detection device, system, and method. The tests carried out with each prototype showed their value, limitations and possibilities in terms of subsequent development and validation through public health research or clinical research. We recognize that a longer-term evaluation is required in order to determine whether the prototypes will be used regularly, integrated into cervical cancer screening services and effectively improve access to cytology as a screening test. We conclude that HCD is a useful for design-based prevention in the field of cervical cancer. The integration of this approach with public health research would allow the generation of evidence during to the formulation of policies and programs as well as optimize existing interventions and, ultimately, facilitate the scalability and financing of what actually works.

## Introduction

Cervical cancer currently ranks fourth among cancer types and is the fourth most common cause of cancer-associated death worldwide [1]. In 2018, the mortality rate due to cervical cancer rose by 6,9 per 100,000 women compared to the previous year, with 311,365 deaths in total. By 2030, it is estimated that there will be a 21,3% increase in incidence and 26,7% in mortality [2]. In Latin America and the Caribbean, the mortality rates are three times higher than in the United States [3], which reflects greater barriers to healthcare in low-income and middle-income countries.

Traditional public health approaches to cervical cancer prevention reported in Latin America have been focused on screening and health education; and in recent years, in vaccination against Human Papillomavirus (HPV) [4,5]. Countries such as Cuba [6], Venezuela [7], Mexico [8], Brazil [9] and Colombia [10] have implemented educational guides to modify prevention-related knowledge and behavior. However, these countries have argued that there is a need to strengthen secondary prevention strategies for the early detection and timely treatment of women at risk. In turn, a series of studies in the region have concluded the need to carry out cytology-based screening differentially, considering the characteristics of the population groups to be intervened [11–17].

Despite advances in early detection, scientific literature shows that Latin American women continue to experience barriers [18]. Structural barriers include the weakness to implement primary care by health services, low quality of preventive programs and processing of laboratory results, deficiency in information systems, and low training of health services providers to monitoring cases of women with precancerous lesions [10,19–22]. Individual barriers include lack of knowledge about cancer and its screening methods, fear of invasive procedures, attitudes of fatalism, religious or spiritual beliefs, concerns over confidentiality, perceived discrimination, embarrassment, partner disapproval, and women's lack of adherence to preventive programs [23–25].

This shows that it is necessary to develop renewed forms of prevention that allow improving women accessibility to prevention programs, especially those with low incomes. An opportunity to generate these innovations is in the field of Human-Centered Design (HCD) that initially originated in the areas of ergonomics, computing and artificial intelligence while trying to make systems more interactive and usable by people [26]. Currently, HCD prioritizes the behaviors, meanings, needs, experiences and values of people, and sets a framework to move rapidly into action [26–28].

The importance of applying HCD in the health sector is being progressively embraced. Health design is a relatively incipient area of research and development of an interdisciplinary nature where the fields of design, health, engineering, physiotherapy, occupational therapy and social research converge [29]. Design companies, healthcare organizations, startups and innovation networks (such as IDEO, Kaiser Permanente, Mayo Clinic, Omada Health, Center for Care Innovations) are using HCD to design innovations such as medical devices, patient-centered experiences and medical healthcare systems.

In public health and more specifically in cervical cancer prevention, HCD can improve the commitment of the community; streamline both the identification of challenges and the design and implementation of programs that tackle complex issues [27].

Stakeholder and key community engagement is critical to effectively transfer to the real context. Communities and members who would implement innovations must become active partners in its refinement during design. Thus, the results of the communitarian co-design are useful for complex problems solutions analysis and development, with longer-term impacts [30]. A scoping review [31] published in 2017 revealed 21 studies where HCD was applied in areas such as disease management, serious and chronic health conditions [32–35], health systems and care management [36,37] infectious disease prevention/care [38,39], and primary prevention and health behavior/education [40,41]. In this review, the authors conclude that there are generalized problems in terms of the description of the applied methodologies as well as the evaluation of the effectiveness and lifespan of HCD-based projects. The developments were carried out in the United States and countries in Western Europe and Africa; there was no evidence of the application of HCD in Latin America nor in the cancer prevention field.

In Colombia, cervical cancer is the fourth cause of death by cancer in women, with an incidence rate of 12,7 per 100,000 women and a mortality rate of 5,7 per 100,000 women [2]. The cytology-based screening strategy has been used for its prevention in the past three decades; however, as in most Latin American countries, national data reveals the persistence of barriers in the access to this test, a lack of follow-up on precancerous lesions and obstacles for definitive diagnosis and treatment [21,42–44]. In this country, the prevention of cervical cancer has become a complex issue that healthcare services have not been able to solve with traditional public health strategies within a mixed public/private healthcare system, based on the insurance and subsidy to the poorest population. Although the official data states that health coverage extends to 95% of the Colombian population, there is sufficient evidence to indicate the presence of barriers in the accessibility to diagnosis and treatment with opportunity and quality [45–48].

Catering to these needs and in the context of the macro-project titled ‘Strengthening accessibility to preventive services in primary care centers in Cali, Colombia*’*, we formed an interdisciplinary team with the purpose of contributing to the improvement of access to cervical cancer prevention using the HCD. We sought to truly involve women, communities, managers and providers of healthcare services in the co-creation of the proposals. In this article, we present the results of the first phase, centered on the design of functional prototypes, proposed by HCD as a method to identify and refine promising ideas aimed at innovative solutions [27]. In this process, we carried out rapid tests to integrate the lessons learned and establish a solid solution based on the macro-project phases. The results of the following phases, which have yet to be published, include the validation of some of the designed prototypes, and the evaluation and redesign of the cancer program implemented in primary healthcare centers in Cali, Colombia. The implementation meant starting slowly and iterating through the HCD for the continuous improvement, broadening and sustainability of healthcare services.

## Materials and methods

This study is linked to the network SUGAR (https://sugar-network.org/network) composed of universities around the world such as Aalto University (Finland), Trinity College Dublin (Ireland), Swinburne University of Technology (Australia), École des Ponts ParisTech (France), University of St. Gallen (Switzerland), Kyoto Institute of Technology (Japan), University of Sao Paulo (Brazil), among others. Since 2007 the Pontificia Universidad Javeriana Cali, Colombia through a global interdisciplinary course on HCD (ME310) has participated in the creation of more than 30 new products and services that have been developed for international companies such as Autodesk, Kodak, Panasonic, Tupperware, Telefónica, Yanmar, Valeo, Philips, and Berg, and for Colombian companies such as Belcorp, Banco de Occidente, Carvajal, Forsa, Totto and Grupo SURA [49,50].

This article presents the application of the HCD in the improvement of preventive services for cervical cancer provided by primary health care centers. The project was developed in collaboration with the ‘Red de Salud Ladera–Empresa Social del Estado (ESE)’ in 38 urban and rural public health centers. In 2018, this healthcare network had a registry of 174,774 women between the ages of 25 and 65 years. The participants were women that were scheduled to take the cervical cytology, women that had never taken it, healthcare providers of the cancer program, the healthcare centers administrators and the general manager of the network.

Research team consisted of two public health experts with experience in health services research in Colombia, a leading HCD practitioner in ME310, two clinicians with experience in detection and care services for women with cervical cancer, two experts in computer science and electronics and a graphic designer. Data collection process was also supported by two Master’s in Public Health. The study was reviewed and approved by the Ethics Committee of the Faculty of Health Sciences of the Pontificia Universidad Javeriana Cali, Colombia. All participants in each group (women, providers, administrators and manager) signed the respective informed consent according to the stage or prototype test of which they took part. The individual in this manuscript has given written informed consent (as outlined in PLOS consent form) to publish these case details.

### Human-Centered Design (HCD)

HCD is a process used to gather information regarding the needs of the beneficiaries of an innovation, create innovative approaches to meet said needs, and offer solutions that work in specific socio-economic contexts [51,52]. Its principles are a design driven and tuned through user-centered evaluation; a process with an iterative nature; completely grasping the experience from users, and building a design team with interdisciplinary skills and perspectives [53].

Literature has reported tension between the way HCD research is carried out versus traditional health research, mainly affecting the reporting and publication of reports [31]. However, several authors have pointed out that the way in which HCD has been disseminated should not be confused with the rigorous primary literature that shows the architecture of its theoretical and methodological foundations [54–58]. This is why there are authors who argue that although HCD has been a relatively new practice for health research community, it is valuable if it is considered as a flexible but disciplined approach to innovation that prioritizes people needs [59].

In our case, the purpose was to move away from the traditional forms of cervical cancer prevention, focusing on the needs of all parties involved in accessibility to cytology, as the main screening test in primary healthcare centers. This is how our methodological route integrated HCD with public health research, particularly with health services research. For a proper dissemination of our work in peer-reviewed literature we follow both the COREQ guidelines [60] for reporting of qualitative research and the reporting guidelines of health research involving design [61].

This project is comprised of five stages: **i.** Research, **ii.** Need synthesis, **iii.** Ideation and co-design process, **iv.** Prototyping and **v.** In context usability testing. To data collection, we used observations, open-ended interviews and conversations, multi-stakeholder workshops, surveys, focus groups, systematic text condensation analysis and tests in real context and simulated hospital. In practice, the stages overlapped and iterated throughout the design process developed between January and December 2018. In the first six months, we carry out the initial 3 stages and in the next six months we prototyped and performed usability testing.

### Research

We carried out participant observations in all the public health centers of the ‘Red de Salud Ladera’ (n = 38). Initially we observe geographical barriers, physical accessibility and infrastructure for healthcare service delivery. Next, we registered observations from waiting rooms, administrative offices, consulting offices and available equipment and inputs for cervical cytology. The total hours of observation were 114 and were carried out by the design team and personnel previously trained in the participant observation technique. We recorded the observations using field notes detailing the general context and cervical cytology provision in each healthcare center.

On a subsequent moment, we carried out open-ended interviews with women for which we used a purposive sampling with volunteers classified into two groups: women with and women without cytology in the last six months. We interviewed women with cervical cytology in the waiting rooms of the healthcare centers (n = 35). We used an interview guide with prior content validation by expert judgments seeking socio-demographic information, knowledge on cervical cancer and cytology, and the perception of the experience before, during and after taking this test. In turn, we identified women without cytology in the databases of the health centers, contacted them by telephone, interviewed them in the health centers with previously agreed appointments and explored the reasons why the Pap test had not been performed (n = 18). All the women interviewed were in an age range between 18 and 71 years, mostly housewives and informal workers.

On the other hand, we conducted open-ended conversations guided by discussion guides with healthcare providers (doctors, nurses, auxiliary nurses) and administrators of healthcare centers selected by convenience sampling. In these conversations we explored their general perception of the cervical cancer prevention program, the motives expressed by women to avoid taking a cervical cytology, how to build a relationship with patients during the test, and the follow-up process on the delivery of results, diagnosis and treatment. All the interviews and conversations were audio recorded for later transcription and analysis.

To complete this research stage, we carried out a benchmarking on the internet with the purpose of finding ways to respond to challenges similar to the one proposed in this project. Hence, we performed a systematic search in Google of experiences that could serve as inspiration in terms of products, services and processes to improve access to healthcare services. The search was guided by keywords in Spanish and English: ‘HCD’, ‘cervical cytology’ and ‘healthcare services’. First, we identify the websites to investigate and then we navigate and analyze each site. This search was systematized by means of an evidence matrix; information extracted from the relevant experiences included the following: country, organization/company and a summary of the experience. With this matrix, we entangled discussions with healthcare providers. In the end, we collected insights from the experiences “Generación Más” of the Ministry of Health and Social Protection in Colombia, Diva Centers in Zambia [52], the Adventure Series in Pittsburgh, United States [53], the video-based visitation system at Mayo Clinic in United States [62], and the InstaPap device in Mexico [63].

### Need synthesis

In this stage, we held a multi-stakeholder workshop with the doctor coordinator of the cancer program, the administrator of one of the healthcare centers, the manager of the healthcare network and the design team. This workshop allowed us to validate the topics identified in the previous stage and the results were illustrated in three different forms: **i.** Persona archetypes, **ii.** Journey maps and **iii.** Areas of opportunity. The archetypes were fictitious characters built from previous research stage; we defined four archetypes of users of the cervical cytology; uninformed women, fearful women, women with no previous cervical cytology and women adhere to the cancer care program. Journey maps are visual representations of the different moments of interaction of women with the cervical cytology service, which could prove useful to redesign the overall patient experience. In terms of the areas of opportunity, we posed the question “how might we improve access to cytology?” to generate ideas in the next stage.

In order to synthesize user needs, we used Malterud’s proposal “systematic text condensation” [64], which is theoretically based on Giorgi’s psychological phenomenological analysis [65]. From this perspective, the essence of phenomena is sought by critically attending to the voices of those who live the experiences, and then discriminate them precisely in units of meaning. Our analysis was carried out by four team members. First, we had an overview of the interviews transcript and looked for preliminary topics. At this time, we express our interpretive and intersubjective positions to focus on women's voices regarding cervical cytology, and not on our own preconceptions as a design team. As a second step, we use Atlas ti software, v8. and we start coding the data in relation to previously defined topics. Here, we took into account the frequency of the codes and identified recurrent patterns. Then we condense the codes into units of meaning and finally we condense the information descriptively.

To ensure data validity and reflexivity, we carry out two types of triangulation [66]. A researcher triangulation where the analyzed data report was reviewed by three of the design team participants with different abilities and disciplinary background in the development of innovative solutions from HCD. Then, we performed data source triangulation comparing the report and identifying common elements between the analysis of the interviews and the field notes that resulted from the open-ended conversations with healthcare providers and administrators of healthcare centers.

The final analysis allowed us to determine five essential needs to consider to improve accessibility to cervical cytology:

- Strengthen education on cervical cytology and HPV.
- Promote and offer the use of cervical cytology to women who have never used it.
- Modify the speculum, perceived as an instrument that causes fear and discomfort.
- Delay in delivery times of cytology results with risk of loss of women to follow up abnormalities.
- Harness the time spent by patients in the waiting rooms.

### Ideation and co-design process

**Fig 1:**
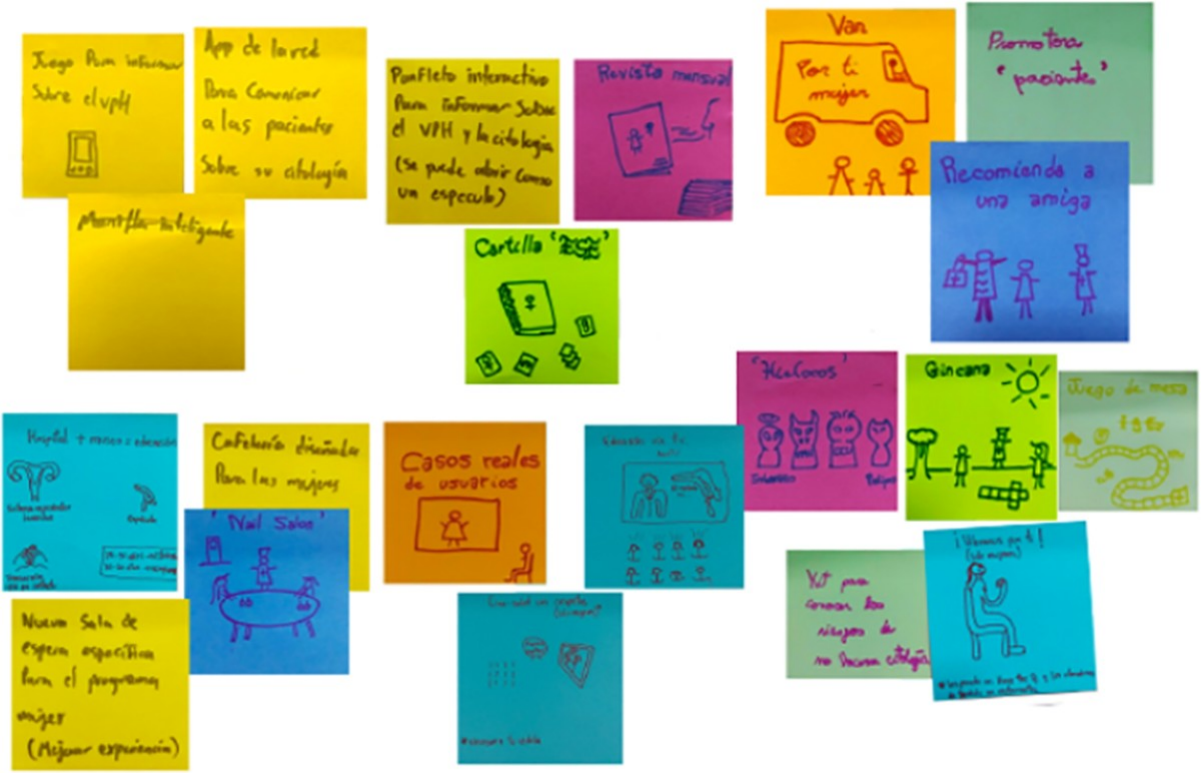
Brainstorming during ideation and co-design stage.

The ideation and co-design process was carried out with the participation of both women users of the cervical cytology service and healthcare providers of the cancer program, healthcare center administrators and the general manager of the health network. With this we ensure the balance of power differential during the generation of new ideas. First, we conducted 15 workshops with 9 multi-stakeholder. In these workshops we used multidisciplinary ideation and each participant presented their points of view to propose solutions to the problem posed [59]. In this way, we generate the highest amount of possible ideas through creativity techniques (e.g. brainstorming, blue slip, list of attributes, brainwriting) [67] (Fig 1). The number of ideas were narrowed down and, at the end of the process, each participant voted on the concepts that they would like to see built through prototyping. Second, we held 8 implementation meetings, where the women gave feedback on the application of each of the prototypes with usability tests in context.

Formal data included field notes of the 15 co-design workshops and 8 implementation meetings. Informal data was also gathered, including notes taken from conversations with the community and photographic records during the observation periods.

### Prototyping

A prototype is an early sample of a product or service before being used in real context [68]. Prototyping gives us the opportunity to test an idea, experiment and reduce uncertainty in innovation processes [69]. This means taking a technical test (if we are talking about a product) or an experience test (if we are talking about services) as close to reality as possible to respond to the needs of end users [70]. Some authors have proposed six types of prototypes: quick and dirty; critical-functional, critical-experience; darkhorse (unexpected concept); funky (near the end); and proof-of-concept (comprehensive proof of concept) [71]. The first prototypes serve to test an idea with real users in their own context. Subsequently, they serve to validate a function, verify its technical feasibility or to test extreme ideas that break schemes and awaken the imagination.

In this stage we design four critical prototypes. During a design team workshop prior to in-context testing, we evaluated the viability of their implementation in primary healthcare centers, the usability and alignment between the needs of women in terms of accessibility to cervical cytology and the interests of the healthcare network compared to their goals regarding screening coverage, opportunity and quality of care.

### In-context usability testing

After refining each prototype in the previous stage, we tested their usability in the waiting rooms of the healthcare centers, in the community environments of urban and rural areas where these centers are located and, in the laboratory of a simulated hospital. It is important to point out here that we carry out prototyping and context testing in an iterative and participatory way. Each prototype received feedback and the experience was properly tested as we explained below.

## Findings

The following four final prototypes emerged from this process.

### ‘Encanto’: Educational manicure

**Fig 2:**
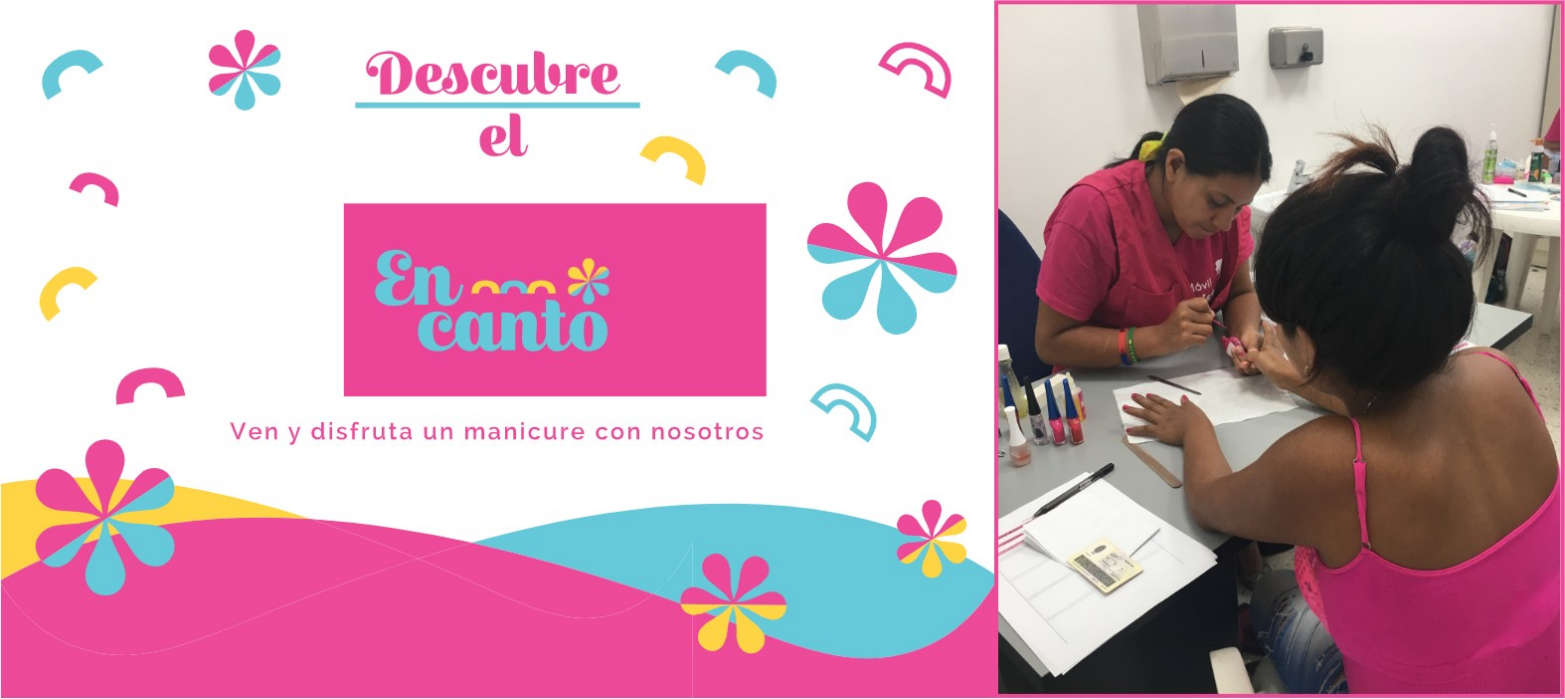
Prototype ‘Encanto’: Educational manicure. Cali, Colombia, 2019.

Given the culture of the city of Cali and the importance of personal image for women, we implemented a first critical-experiential prototype called ‘Encanto’, a free service of educational manicure in the healthcare centers waiting rooms (Fig 2). The experience was developed in alliance with a local business called ‘La Manicurista’, consisting of an App offering manicure service at home. Before implementing ‘Encanto’, we trained the manicurists staff in cervical cancer prevention education. In one of the healthcare centers, we customized the area and displayed informative material inviting women nearby. During the manicure, the educational process was carried out, which included information on the disease, its early detection through Pap test and HPV tests and a review and demystification of wrongful ideas regarding the disease. When the manicure concluded, we invited women to take the Pap test.

Testing of this prototype included non-participant observation of the interaction between women and manicurists as well as the application of a perception survey to 15 women (20 to 62 years; housewives, employees, informal workers and a technical school student). The survey contained three questions:

- What is your opinion of the 'Encanto' experience?
- How did you find the information provided by the manicurists regarding cytology?
- After the 'Encanto' experience, what do you think of cytology?
- Among the responses to this survey we highlight the following:

“*It's rare but cool to find a nail salon here at the hospital*. *So you don't waste time in the waiting room doing nothing*, *and it is very good that they give you information about cytology*” (Women, aged 48)

“*The lady who did the manicure showed that she knew of cervical cancer*. *It is good to share what you think about cytology with another woman*. *She clarified many things for me that I had not discussed with the doctor for shame*. *I hope this continues to be done here in the hospital*” (Women, aged 26)

Through verification in the health center database and phone calls, we found that of the 15 women who participated in the ‘Encanto’ experience, 12 underwent cytology during the following week; 3 women did not do it arguing lack of time due to childcare.

We analyzed the results of the ‘Encanto’ test in a design team discussion group. Our insights and learnings for subsequent validations of the prototype on a larger scale were: **i.** in the context of local culture, ‘Encanto’ was an experience valued positively by women, who highlighted taking advantage of the waiting times through education in health; **ii.** Given that, ‘Encanto’ was oriented to women in poverty-stricken settings, the free nature of the service was key to motivate participation; **iii.** Its implementation must assure prior training of the staff giving the manicure regarding the prevention of cervical cancer. **iv.** Finally, due to the appeal of the service, the healthcare center must attend to the demand induced by ‘Encanto’ and schedule Pap test appointments in a timely manner.

### ‘No le des la espalda a la citología’: Media-based strategy using YouTube, social networks, SMS and Beacon devices

With the purpose of improving accessibility for women that have never taken a Pap test and rarely attend primary healthcare facilities, we conceived the second critical-functional prototype as a media-based strategy called “No le des la espalda a la citología” (‘Do not turn your back on cytology’).

This strategy consisted of:

- A YouTube channel. We launched a campaign using the hashtag #ValePrevenirHoy (It is worth to prevent today) in social networks: Facebook, Twitter and Instagram; in YouTube, we posted an educational video for two months and a half. We obtained 119 visits, exceeding twice the visits of the official channel of the healthcare network in the same period.
- WhatsApp and Short Message Service (SMS). We floated the video around the internet through WhatsApp and SMS among women with no cytology report. From a database of 9,529 women with a valid cellphone number, 246 messages were randomly sent to users, from which 60% were effectively read. Out of the 200 SMS sent, 98% were read.
- Bluetooth message through Beacons. Beacons are devices that broadcast messages using a low-cost Bluetooth connection; their focus lies in proximity marketing, which is the wireless distribution of advertising content on a local scale with commercial purposes. We took advantage of this technology and spread the previously mentioned video by placing Beacons in a healthcare network, a central park in the urban area of influence of the healthcare network, and a mobile unit that roamed through rural areas. After analyzing the data, it was determined that, although there were 297 message notifications in the targeted cellphones, few people had effective access to the video.

The iterations carried out with this prototype and its results were analyzed by the design team in discussion groups in which the manager of the healthcare network participated. During the iterations we realized the need to refine the educational messages contained in the video, emphasizing the free nature of cervical cytology and consultation without an appointment in all the health centers of ‘Red de Salud Ladera’.

Our insights and learnings for larger scale prototype validations were: **i.** it is necessary to articulate media strategy to the official communication channels of the Health Network; **ii.** Of the three media used (YouTube, WhatsApp Messaging and SMS and Beacons), reading SMS messaging had a response of 98%, making it the means of communication with the greatest reach in the population studied; **iii**. in the voice of the manager of the healthcare network, the use of Beacons with preventive messages to promote accessibility to cervical cytology is a low-cost tool, easy to implement, with the potential to reach women quickly and widely in different contexts of daily life and outside health centers. However, these messages should be short, direct and without links to other web sites such as videos. In this regard, the manager commented:

“*I'm interested in applying Beacon technology to reach people who hardly ever go to health centers*. *I can circulate mobile primary care health units to send concrete messages to the community*. *This would imply that when we arrive at the health centers we ask the women if this type of message reached their cell phones and if that is why they come to cytology*” (Healthcare Manager, ‘Red de Salud Ladera ESE’, Cali, Colombia)

Finally, **iv.** this prototype led us to conclude that, in order to value the effectiveness of the media-based strategy, a follow-up mechanism must be included that can identify the women outreached with this technology and, subsequently those who visited primary healthcare centers to take a Pap test.

### ‘Tu Turnero ESE’: Educational wireless queuing device

**Fig 3:**
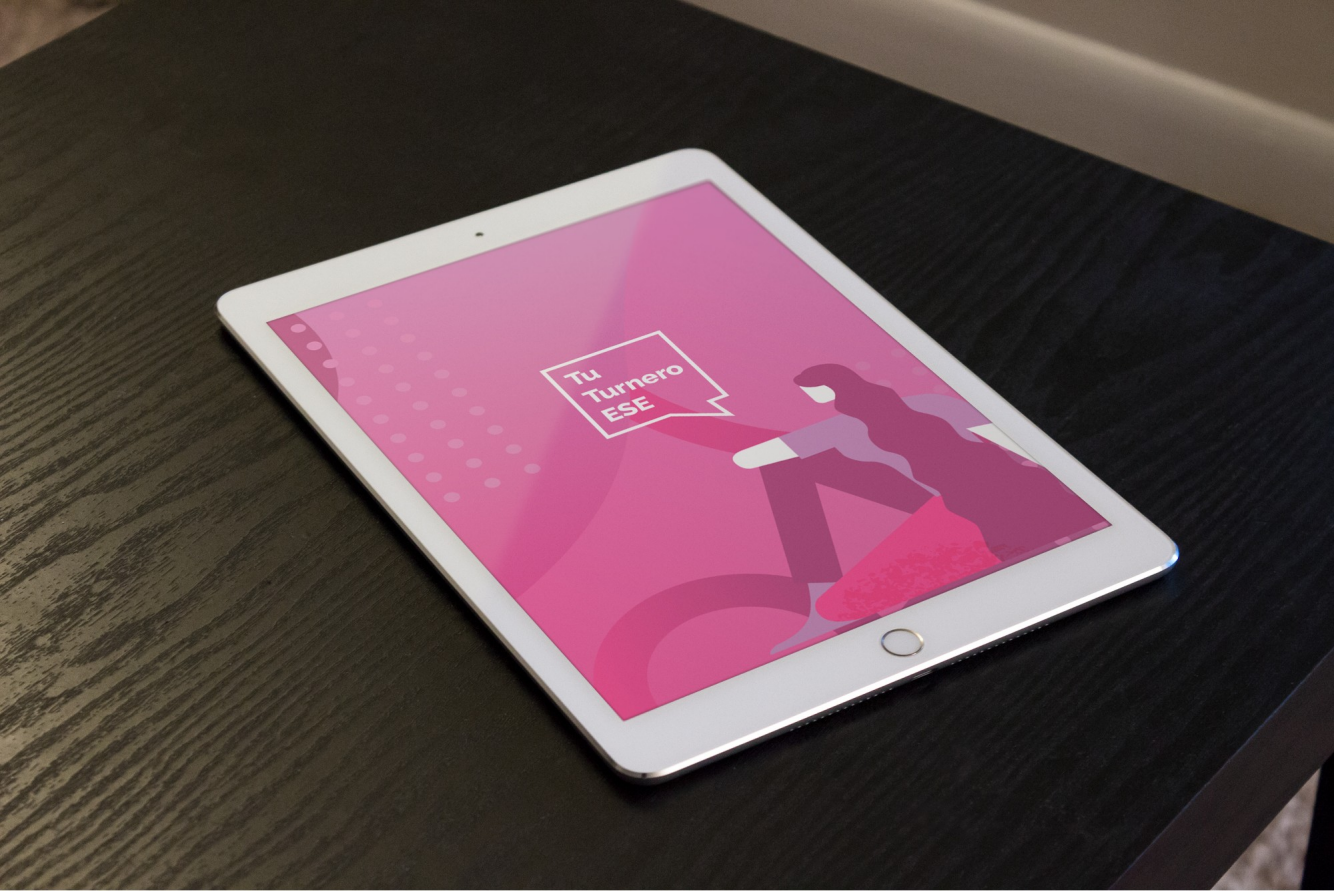
Prototype ‘Tu Turnero ESE’: Educational wireless queuing device. Cali, Colombia, 2019.

Seeking to optimize time in the waiting room, we designed an educational App embedded into a tablet (Fig 3). This new App also served as a queuing device. The app included a risk survey for cervical cancer and prompted women to take a Pap test based on their result. The survey inquired on their knowledge, attitudes and practices; each question, according to the answer (YES or NO), was followed by an informative header that reinforced healthy behaviors or clarified answers deemed to be incorrect or unsatisfying for the use of cytology cervical. The final message of the survey encouraged women to take a Pap test. All intervention content was designed to be consistent with the American Cancer Society recommendations and Colombian guidelines for cervical cancer care [72,73]. During the experience test, a team member observed and recorded the behavior of each woman in her interaction with the educational ‘Turnero’.

**Fig 4:**
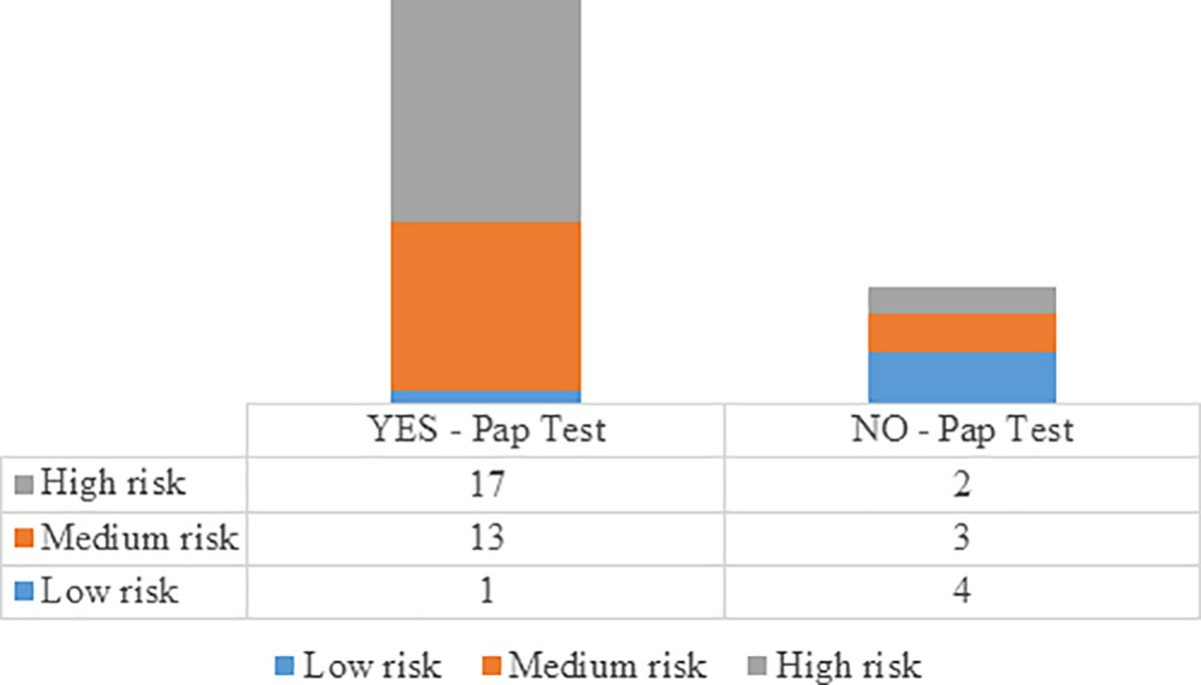
Pap Test/Perceived susceptibility post "Tu Turnero ESE" experience.

The test of this critical experiential prototype was carried out with 40 women (18 to 63 years; housewives, employees and informal workers), 31 of whom underwent cytology after being exposed to 'Tu Turnero ESE'. Detailed results are presented in Fig 4.

We integrated the results of observations, survey and effective use of cervical cytology in a discussion group made up of two women who participated in the experience, three health providers and the design team. Our insights and learnings for larger scale prototype validations were: **i**. Harnessing time in the waiting rooms through an educational queuing device was an experience valued positively by women. They no longer considered that they were wasting time that could be spent in taking care of their children or other household chores; **ii.** The content of the app was appropriate in terms of the average waiting times of healthcare centers; **iii.** The tablet was not an adequate tool for women over 60 years old, who had difficulty handling a touchscreen. In this regard, we highlight the following statements:

*“It is always good to take advantage of the time while you wait for the doctor to attend you*. *So one does not feel so guilty about leaving the children at home*. *I liked the tablet and the survey*, *it really got me thinking about cytology*, *I learned things I did not know and so*, *knowing*, *cytology becomes easier*” (Women, aged 37)

*“I think the important thing is for women to be aware of their risks*. *In this sense*, *if the survey incorporated into the educational Turnero causes cytology to be performed*, *I consider that we must apply the strategy in all our health centers*, *especially with women willing to handle the tablet”* (Healthcare Provider)

### Citobot

We initially designed the Citobot (Cervical cancer early detection device, system, and method, provisional utility patent application number 62855125 / MAY-31-2019) by identifying that the discomfort caused by the speculum can deter women from taking a Pap test. This procedure is often seen as a painful experience, thus reducing the willingness to retake said test. Furthermore, the delivery times of the results varied between 20 to 30 days, significantly delaying the opportunity for subsequent diagnosis and treatment.

**Fig 5:**
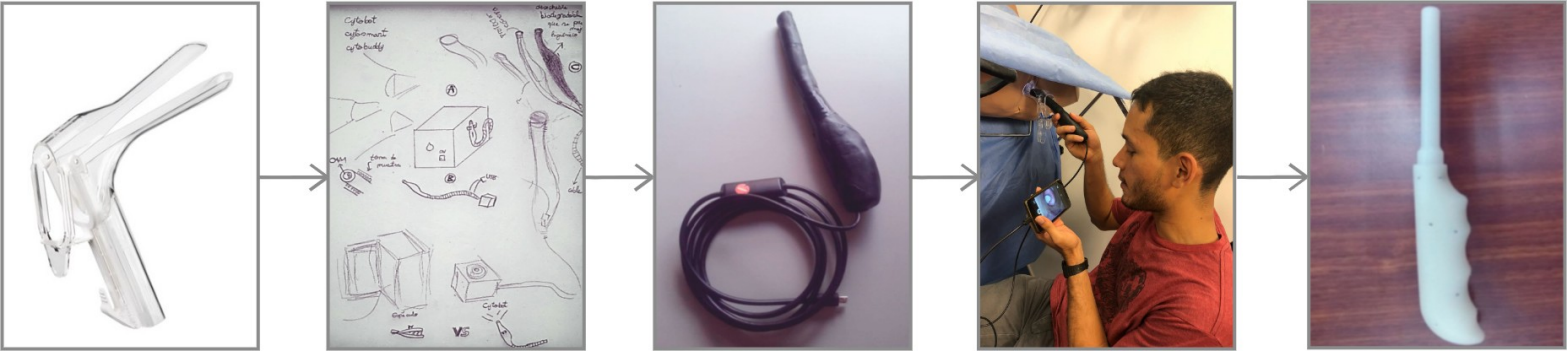
Designing and testing Citobot. Cali, Colombia, 2019.

The Citobot is a critical-functional prototype, designed as an alternative to the speculum and with the intention of detecting precancerous lesions in an immediate manner using an artificial intelligence system. Built using the computer-assisted design (CAD) software SolidWorks, the device uses an endoscopic camera to display images taken from the cervix in real time. We illustrate the design and testing sequence in Fig 5 (from left to right, 1. speculum, 2. original sketches, 3. first prototype, 4. testing in simulated hospital, 5. second prototype)

We performed two types of tests. First, in a simulated hospital with a pelvic Gyn/Aid ® model with different types of cervixes (healthy cervix, linear laceration, cervical erosion with mucous, cervical polyp, Nabothian cyst, purulent acute cervicitis, and carcinoma), where the design, camera lighting, resolution and angles of view of the cervix were validated. The test allowed to verify the Citobot operation and compare it with the speculum. It was found to be taking sharp pictures of the cervix. However, the speculum's viewing angle was greater than that of the Citobot, so this device requires further modifications in hardware development. Second, we conducted interviews with 15 women (22 to 51 years; housewives, employees and informal workers), two doctors, two gynecologists, and two biomedical engineers. The interview with the women explored the general perception of the Citobot and the acceptability for its use. This device is a prototype perceived by women as a promising alternative in comparison to the speculum, turning the Pap test into a more comfortable experience. Doctors and gynecologists valued the huge potential of the device in the reduction of result delivery times and, hence, the rapid detection of precancerous lesions. Comments in this regard were:

*“The Cytobot is a thousand times better than the speculum*! *due to its shape*, *it should be much more comfortable*. *I don't think there is a woman in this world who likes speculum*! *this is much better and if it helps to be diagnosed quickly*, *the better*!*”* (Women, aged 33).

“*The big problem we have in Cali and I would say that in Colombia in general is the delay in delivering cytology results*. *If the Citobot allows us to detect precancerous lesions during the consultation and we can refer women immediately to colposcopy to verify the diagnosis*, *it would be great*! *I think it has great potential as a medical device to expand screening coverage and that we can effectively prevent cervical cancer mortality”* (Gynecologist)

**Fig 6:**
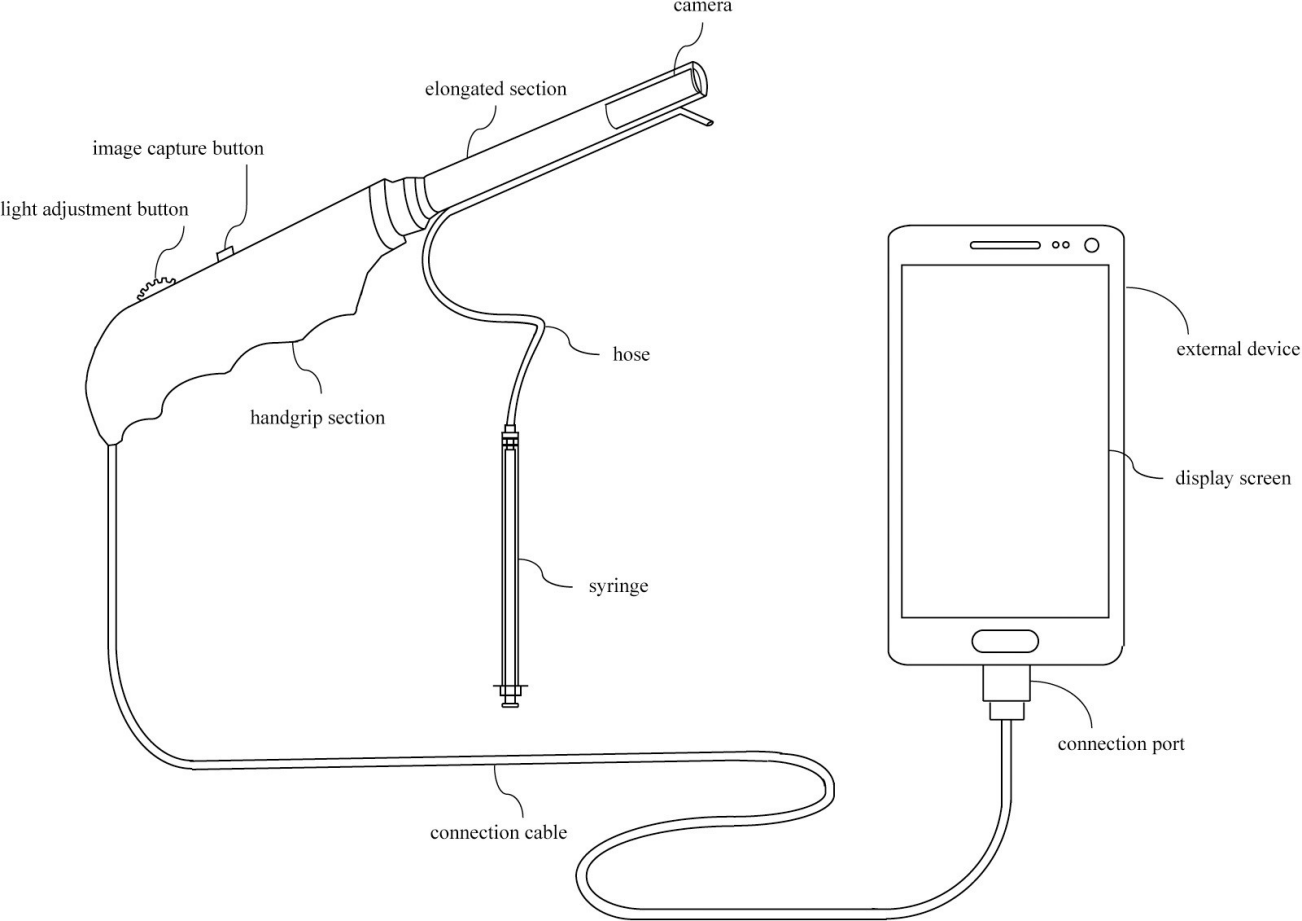
Citobot 1.0, cervical cancer early detection device, system, and method.

The Citobot (Fig 6), as a portable electronic device, is comprised of two sections i. an elongated slender section covered by a disposable sheath which contains a camera inside of the end of said section, ii. a thicker section serving as a handgrip with buttons to capture images with the camera and adjust the intensity of the emitted light. The observation of the cervix involves the camera connected to an external display device (cellphone, tablet or computer). Additionally, the device includes an acetic acid injection mechanism to achieve the required coloration of possible lesions in the cervix.

The artificial intelligence (AI) system works as follows:

- Image capture: An image of the cervix is captured with the device and sent to the AI system.
- Image pre-processing: A pre-processing stage is required prior to the analysis, seeking to facilitate the comparison of the image with a previously defined database. This stage corroborates whether the image can be analyzed by the system due to factors such as excessive blurriness, insufficient lighting or a tarnished camera lens.
- AI-based analysis: The image is processed by the artificial intelligence system, which is trained beforehand using a database comprised of images of cervixes.
- Preliminary assessment of the level of risk: The AI system determines the level of risk of cervical cancer (low or high) and displays a notification to the healthcare practitioner.

The National Department of Science and Technology in Colombia, COLCIENCIAS have benefited Citobot in 2019 for the procedures required by the International Patent System (Patent Cooperation Treaty–PCT).

## Discussion

Cancer is a dominant disease worldwide and premature deaths occur specially in low-income and middle-income countries such as Colombia [19]. This article presented the design process of innovative prototypes for cervical cancer prevention in primary care centers located in low-income settings in Cali, Colombia using HCD. This useful methodology allowed us to acquire information and propose four prototypes with the potential of improving access to the Pap test. It focused on the needs of women and the organizational characteristics of a primary healthcare network, and their area of influence in socioeconomically vulnerable communities.

In other contexts and experiences [74,75], HCD has proven its value in the development of programs and even policies [76]. The literature discussing its application is abundant in many areas, as well as reports of experiences such as the reorganization of clinical installations [62], the reduction of medication errors [37,77], and cancer care [78,79]. In the field of healthcare services, HCD has the potential to reduce the “knowing-doing gap” [80] to transition from traditional operation methods to an approach centered in co-design with the communities involved and thus respond more accurately to their needs [30]. More concretely, prototyping can be used to carry out quick experiments and iterations that contemplate human factors, which ultimately rule the adoption, acceptability and compliance of healthcare programs [81].

Application of HCD in the prevention of cancer in general and cervical cancer in particular has been limited. In 2014, research carried out with executives from payer groups and health care delivery systems, health care providers, and adult patients revealed that this transdisciplinary methodology had not been commonly used for quality improvement efforts in cancer care [82]. In this same sense, Bazzano and colleagues review of studies conducted between 2006 and 2016 [31] found only one HCD-based research that aimed to design a workbook ('The Families SHARE') for cases of breast cancer, colorectal cancer and other diseases. The results of this study were especially useful for disseminating family risk information and encourage risk reducing behaviors [33]. In our case, we have presented in this article an initial contribution to the field of cervical cancer early detection with HCD-based methodology and its corresponding design and rapid tests in the context of critical-experience and critical-functional prototypes. Two of the prototypes were aimed at the inner setting of the healthcare centers ('Encanto' and 'Tu Turnero ESE), a third, sought to attract women in external settings outside the clinics who had not undergone cervical cytology ('Don't turn your back on cytology': Media-based strategy), and the fourth prototype (Citobot) seeks to improve the comfort of cytology and reduce the delivery times of this exam results.

Our findings can be partially contrasted with previously used interventions to increase cervical cancer screening from traditional public health approaches. Regarding the ‘Encanto’ prototype: educational manicure, the use of one-on-one education recommended by The Community Preventive Services Task Force [83] has demonstrated strong evidence of effectiveness in increasing screening [8,84–86]. 'Por la Vida' model [87], ‘Las Mujeres Saludables’ (Healthy Women) [88], ‘Cultivando la Salud’ (Cultivating Health) [89] y ‘Entre Amigas’ (Between Friends) [85] have been programs implemented in the United States with low socioeconomic status Latina women that have been shown to be effective and viable interventions to increase the use of cancer screening tests. As in ‘Encanto’, these interventions used ‘Consejeras’ or ‘Promotoras de Salud’ (i.e., Latina health advisors) to carry out culturally appropriate education. In our case, manicurists became models, as indicated in the theory of social learning [90], at the time of education during manicure sessions. In subsequent validations of this prototype, it is recommended to evaluate health outcomes such as Pap test knowledge and self-efficacy, perceived susceptibility, perceived benefits of having a Pap test and perceived survivability of cancer.

Two additional elements make ‘Encanto’ an innovative prototype that we would not have achieved using a traditional approach to health education. HCD allowed us to quickly identify two community needs and provide a synergistic solution. First, we detected the poor perception of women about waiting times in health centers, and second, we identified their interest in manicure rooms, which corresponds to the cultural pattern of the city of Cali where women, regardless of their socioeconomic level, prioritize the care of their personal image and beauty. In this way, we decided to bring manicure as a motivational factor to the waiting rooms of health centers. It should also be noted that ‘Encanto’ was an experience test of interest for women in all age groups, unlike, for example, the ‘Cultivating Health’ intervention, where only women who were older than 50 years participated. As our results showed, ‘Encanto’ was an educational experience valued positively by women; however, in the future, both its effectiveness in improving accessibility to the Pap test with larger samples of women and the feasibility of its implementation in the organization of cervical cytology services should be evaluated. Issues like manicure funding, the provision of physical spaces in waiting rooms, and the coordination of waiting times with medical care should be reviewed.

Regarding our second prototype ‘Don't turn your back on cytology’: Media-based strategy, our findings also supports the Task Force [83] recommendations for use of client reminders, or small media materials for increase demand for screening services. The use of m-Health understood as the use of mobile and wireless devices to improve health outcomes, health care services, and health research, can lead to behavior change interventions in people who have a phone or a smartphone, but who do not have access to a computer, as in the case of the women in our study. Literature has also indicated that m-Health interventions may be cheaper to implement at population level [91]. We found that more women were reached via SMS than through the YouTube campaign with educational video, confirming the relatively low use of web-based interventions [92,93]. Although the overall impact and reach of SMS interventions on cancer screening are unknown, results of a systematic review published in 2017 reveal how text messaging interventions appear to moderately increase screening rates for cervical cancer [94,95].

Regarding the use of Beacons as health information technology to disseminate our media-based strategy, we did not found previous interventions in cervical cancer screening with which to compare our findings, which represents a limitation for its discussion. The incorporation of the Beacons to the prototype occurred thanks to the HCD methodology with the interdisciplinary conformation of the design team. From a merely traditional perspective of health education we would not have incorporated proximity marketing with Beacons to incentivize the Pap test. The use of this technology is promising to reach women outside of health services in urban and rural settings. Using SMS or Beacons would mean conducting studies that associate message reception with cancer screening behavior. In Colombia, it is also required that healthcare centers are organized in terms of human talent, infrastructure, hardware and software and interoperability between information systems; the country has the potential for this given the expansion of the capacity of telemedicine services that can lead massive prevention campaigns [96].

Our third prototype was ‘Tu Turnero ESE’: Educational wireless queuing device. With this device we achieved the use of time in waiting rooms with cervical cancer prevention education while the women had their turn to be seen by the clinicians. Previous scientific literature has shown that low level of awareness and knowledge about the disease and its early detection are factors that affect the use of Pap tests [97,98], and that health education through lectures, debates, videos and brochures is essential to improve cervical cancer screening [99]. We have incorporated into the wireless queuing device an App that included a risk survey, finding that women with high perceived susceptibility were those who underwent cytology after being exposed to a ‘Tu Turnero ESE ‘. Other studies have already documented, based on the Health Belief Model, that factors such as perceived benefits, perceived susceptibility, perceived severity/seriousness, and perceived barriers median the relationship between cervical cancer knowledge and screening behaviours [100–102].

HCD allowed us to achieve two insights during the design of this prototype. First, ‘Tu Turnero ESE’ required that women actively interact with the device. We were not looking for what other authors have already pointed out regarding the passive consumption of health information in waiting rooms [103]. Second, the risk survey we used was based on 'YES or NO' responses, following recommendations of Waters and colleagues [104]. These authors have explained how respondents who answer 'I don't know' regarding the perceived risk of cancer are less likely to participate in preventive behaviors than people with 'YES or NO' responses. Surveys with this binary format also prevent high rates of ‘don't know’ responses previously detected in sociodemographic groups with limited formal education [104], as the women who participated in this experience. In the future, we recommend for the implementation of this prototype, that healthcare centers provide wireless free internet (Wi-Fi) in waiting rooms and the support of 'Promotoras de Salud' to help older women with difficulty manipulating digital screens, to interact with the App.

Finally, Citobot is a prototype that began its design to respond to fear of pain, discomfort and embarrassment that vaginal speculum examination produces in women; these factors have been identified as barriers to screening [105]. During iterations and simulated hospital tests, we were able to move towards designing a medical device perceived to be comfortable by women, but also with the potential to detect precancerous lesions using an artificial intelligence (AI) system. In this regard, it should be noted that the changes that the speculum has had have been for the purpose of improving external user visualization but have not been thought from the experience and comfort of the patient [106]. Citobot not only provides comfort, but has an utmost importance in the reduction of time gaps for the delivery of Pap test results, avoiding the loss of track of women with precancerous lesions and encouraging timely diagnose and treatment.

Devices similar to Citobot detected in our research stage and benchmarking were: a 'dilating speculum' called the Veda-scope [107,108], the POCkeT Colposcope [106] and the Insta Pap [63]. Currently, these devices have different levels of clinical and tech development. In subsequent studies, we will advance clinical research to determine the acceptability, reproducibility, sensitivity, and specificity, concordance with cytology, and predictive capacity of the Citobot. At the same time, we will develop the machine learning algorithms of the AI incorporated into this medical device.

As final thoughts, it is important to recognize that optimizing the provision of cervical cancer preventive services involves complex decision making, multiple transfers between primary care and specialty care providers, and coordination between health service managers and the cancer care team. We must acknowledge that a longer-term evaluation is required to determine whether the prototypes will be used regularly, be integrated into cervical cancer screening services and effectively improve access to cytology. Now, we agree with other authors [109] that HCD is an iterative approach of a transdisciplinary nature that allows to generate a large number of ideas in an agile way, build tangible prototypes, iteratively co-create with people and generate empathetic solutions with end users. However, while ensuring feasibility on a larger scale, all prototypes require refinement through validations with experimental or quasi-experimental studies in public health or through research designs specific to clinical epidemiology. Also, other issues such as technical feasibility, financial viability and sustainability must be adjusted.

Lastly, it is noteworthy to mention that research in HCD has the challenge to include quantifiable results [31], strengthening itself with local resources [110], and engage decision-makers, community organizations and the academia. Evidence generation through this methodology can partner policies formulation and is essential to avoid causing damages, allow the optimization of existing interventions and, ultimately escalate and finance what actually works [111]. The parties interested in the design-based prevention of cancer may even alter the funding landscape for the generation of said evidence and its broadcasting. Such integrations would enable further acceptance of HCD in health research and, eventually improve healthcare services through innovations that combine the identified user needs with feasible initiatives in specific contexts.

## Supporting information

S1 File (XLSX)

S2 File (PDF)
