# Supplementary material for: Innovative prototypes for cervical cancer prevention in low-income primary care settings: A human-centered design approach
Source: PLoS One. 2020 Aug 24;15(8):e0238099. doi: 10.1371/journal.pone.0238099 (PMC7446804; doi:10.1371/journal.pone.0238099)
Supplement: S2 File — (PDF) [file pone.0238099.s002.pdf]

Electronic Acknowledgement Receipt

**EFS ID:**	36166740
**Application Number:**	62855125
**International Application Number:**	
**Confirmation Number:**	9860
**Title of Invention:**	CERVICAL CANCER EARLY DETECTION DEVICE, SYSTEM, AND METHOD
**First Named Inventor/Applicant Name:**	JUAN PABLO GARCÍA CIFUENTES
**Correspondence Address:**	Pontificia Universidad Javeriana<br>-<br>CALLE 18 #118-250<br>-<br>CALI - -<br>CO +57 3218200<br>dlriveros@javerianacali.edu.co
**Filer:**	JUAN PABLO GARCÍA CIFUENTES
**Filer Authorized By:**	
**Attorney Docket Number:**	
**Receipt Date:**	31-MAY-2019
**Filing Date:**	
**Time Stamp:**	12:41:23
**Application Type:**	Provisional

Payment information:

Submitted with Payment	yes
Payment Type	CARD
Payment was successfully received in RAM	$140

RAM confirmation Number	053119INTEFSW12442100
Deposit Account	
Authorized User	

The Director of the USPTO is hereby authorized to charge indicated fees and credit any overpayment as follows:

File Listing:

Document Number	Document Description	File Name	File Size(Bytes)/ Message Digest	Multi Part /.zip	Pages (if appl.)
1		PPACitobot31052019FULL.pdf	698120<br>6fd8a28c0a5c2dd089c6f8e07ccabd26a26be775	yes	17

Multipart Description/PDF files in .zip description

Document Description	Start	End
Abstract	17	17
Drawings-only black and white line drawings	12	16
Claims	10	11
Specification	1	9

Warnings:

Information:

Document Number	Document Description	File Name	File Size(Bytes)/ Message Digest	Multi Part /.zip	Pages (if appl.)
2	Provisional Cover Sheet (SB16)	sb16_JUANPABLOGARCIA.pdf	2032625<br>a79dec6b7172bbe63cd09f06e126354230d1d490	no	4

Warnings:

Information:

Document Number	Document Description	File Name	File Size(Bytes)/ Message Digest	Multi Part /.zip	Pages (if appl.)
3	Provisional Cover Sheet (SB16)	sb16_ANDRESMAURICIOGUERRERO.pdf	2032619<br>f8c3708d84403e8d7ec9399f02369abe670a6472	no	4

Warnings:

Information:

4	Provisional Cover Sheet (SB16)	sb16_DANIELANEIRA.pdf	2032620<br>a67bb63eb647b98c1b10bc6bfa5fec70f7cc5af0	no	4
**Warnings:**					
**Information:**					
5	Provisional Cover Sheet (SB16)	sb16_GIANNKARLOAGUIRRE.pdf	2032624<br>58191dbc61d5d7c0b96059b04e1fd6c64b377153	no	4
**Warnings:**					
**Information:**					
6	Provisional Cover Sheet (SB16)	sb16_JORGEIVANBOTERO.pdf	2032621<br>ed94679a6491e45616387ba4c45edc7341d3f8e8	no	4
**Warnings:**					
**Information:**					
7	Provisional Cover Sheet (SB16)	sb16_MARCELADELCARMEN.pdf	2032622<br>2f6338d038f4a0a684aa2067b315aa0e61a86d7c	no	4
**Warnings:**					
**Information:**					
8	Provisional Cover Sheet (SB16)	sb16_NATALIAJIMENEZ.pdf	2032620<br>6aac7b22d73022e14cfe85305648526854b5d07f	no	4
**Warnings:**					
**Information:**					
9	Provisional Cover Sheet (SB16)	sb16_PAULACRISTINABERMUDEZ.pdf	2032621<br>2d937b754bf6856fa731d550bda3a0310f87ef15	no	4
**Warnings:**					
**Information:**					
10	Fee Worksheet (SB06)	fee-info.pdf	29056<br>e3e6e0b7c711afa839d8f831da122152758c19f8	no	2
**Warnings:**					
**Information:**					
		**Total Files Size (in bytes):**	16988148		

This Acknowledgement Receipt evidences receipt on the noted date by the USPTO of the indicated documents, characterized by the applicant, and including page counts, where applicable. It serves as evidence of receipt similar to a Post Card, as described in MPEP 503.

New Applications Under 35 U.S.C. 111
If a new application is being filed and the application includes the necessary components for a filing date (see 37 CFR 1.53(b)-(d) and MPEP 506), a Filing Receipt (37 CFR 1.54) will be issued in due course and the date shown on this Acknowledgement Receipt will establish the filing date of the application.

National Stage of an International Application under 35 U.S.C. 371
If a timely submission to enter the national stage of an international application is compliant with the conditions of 35 U.S.C. 371 and other applicable requirements a Form PCT/DO/EO/903 indicating acceptance of the application as a national stage submission under 35 U.S.C. 371 will be issued in addition to the Filing Receipt, in due course.

New International Application Filed with the USPTO as a Receiving Office
If a new international application is being filed and the international application includes the necessary components for an international filing date (see PCT Article 11 and MPEP 1810), a Notification of the International Application Number and of the International Filing Date (Form PCT/RO/105) will be issued in due course, subject to prescriptions concerning national security, and the date shown on this Acknowledgement Receipt will establish the international filing date of the application.
